# Development of a Loop-mediated isothermal amplification (LAMP) technique for specific and early detection of* Mycobacterium leprae* in clinical samples

**DOI:** 10.1038/s41598-021-89304-2

**Published:** 2021-05-10

**Authors:** Nupur Garg, Upasana Sahu, Sudeshna Kar, Farhan J. Ahmad

**Affiliations:** 1 Department of Pharmaceutics, School of Pharmaceutical Sciences & Research, Jamia Hamdard, New Delhi India; 2 Department of Molecular Medicine, Jamia Hamdard, New Delhi India

**Keywords:** Biological techniques, Biotechnology, Diseases, Molecular medicine

## Abstract

Leprosy, a progressive, mutilating and highly stigmatized disease caused by *Mycobacterium leprae* (ML), continues to prevail in the developing world. This is due to the absence of rapid, specific and sensitive diagnostic tools for its early detection since the disease gets notified only with the advent of physical scarring in patients. This study reports the development of a Loop-mediated isothermal amplification (LAMP) technique for fast, sensitive and specific amplification of 16S rRNA gene of ML DNA for early detection of leprosy in resource-limited areas. Various parameters were optimized to obtain robust and reliable amplification of ML DNA. Blind clinical validation studies were performed which showed that this technique had complete concurrence with conventional techniques. Total absence of amplification of negative control DNA confirmed the specificity of this test. Various visual detection methods viz*.* colorimetric, turbidity differentiation and bridge flocculation were standardized to establish easy-to-read and rapid diagnosis. This technique eliminates the lack of accuracy and sensitivity in skin smear tests in patients and the requirement for expensive lab equipments and trained technicians. The technique holds promise for further expansion and has the potential to cater to the unmet needs of society for a cheap, highly-sensitive and robust rapid diagnosis of ML.

## Introduction

Leprosy, also known as Hansen’s disease, is a debilitating dermato-neurological disease caused by *Mycobacterium leprae* (ML). After the introduction of Multi-drug Therapy by World Health Organisation in 1984, attempts were made to curb the disease and several countries declared elimination of leprosy in the successive years. However, since 2019, an emergence of 2, 02, 226 new global cases have been observed, mainly in developing countries^1^. This figure is almost stable since the last decade which shows that a perpetuating reservoir of infection still exists. It indicates that the burden and transmission of this neglected tropical disease is still a major health concern all over the world. Preliminary diagnosis of leprosy is conventionally made by symptomatic observations, like hypo-pigmented patches on skin, ulcers, lesions, loss of sensations, limb and facial deformities^2^. The slit skin smear (SSS) test for acid fast bacilli (AFB) and histopathological examination is done for further confirmation of ML infection^3, 4^. Apart from taking days to obtain results and requirement of lab facilities and skilled technicians, major limitations with these diagnostic methods are lack of sensitivity (at least 10^4^ AFB per gram of tissue is required for positive identification), false negatives and false diagnosis of *Mycobacterium tuberculosis* (MTB). Studies have shown that many patients who show negative AFB count in SSS test are actually positive for AFB load in the granuloma^5^. The genomic and morphological makeup of MTB is highly similar to ML as they share the same evolutionary lineage. This leads to misdiagnosis and mistreatment of patients for MTB instead of ML^6^.

An early and specific diagnosis of ML is, therefore, urgently required to prevent physical damage, morbidity and transmission. Researchers have come up with several advanced molecular methods including detection of specific markers of ML. Antibodies directed against the ML-specific phenolic glycolipid I (PGL-I) and leprosy IDRI diagnostic-1 (LID-1) make the basis of many serological tests^7^. Hooij et al.^8^ evaluated two recently designed field-friendly lateral flow assays (LFAs- the OnSite Leprosy Ab Rapid test and the in-house developed PGL-I up-converting phosphor (UCP)-LFA). PCR techniques and nucleic acid amplification techniques have proven to be great tools for diagnosing ML with high sensitivity and specificity. Recently, Beissner et al.^9^ developed a combined RT-qPCR method which simultaneously uses two PCR assays on different genes for increased efficiency. Cabral et al.^10^ detected the nasal carriage of ML in the household contacts of infected persons by using PCR. Similarly, several PCR and immunoassay based techniques have been explored to develop early and rapid diagnostic tools^11–17^. However, the use of these methods for rapid diagnosis of leprosy is limited due to drawbacks such as (i) lack of specificity, (ii) false negatives due to low bacterial load, (iii) false positives due to co-circulating antibodies from other infections and (iv) requirement of expensive equipments and trained labor. Even the newest detection techniques fail to detect 60% of paucibacillary ML infections^3^. As leprosy is endemic in areas where resources are poor, it is even more imperative to develop a test which, besides being sensitive and specific, is economical, rapid and user friendly.

Isothermal amplification methods are favored over traditional PCR methods for developing cheap, effective and rapid diagnostics as the requirement of expensive laboratory equipments like thermocyclers is eliminated, without compromising efficiency or sensitivity. Hence, isothermal amplification techniques have better scope and potential to be used in rapid diagnostics in developing economies. Over several isothermal-based techniques, the loop-mediated isothermal amplification technique (LAMP) has many applications in the field of point-of-care (POC) testing. It utilizes four to six primers to identify specific regions of the template which can be amplified at constant temperature, using DNA polymerase with high strand displacement activity. Therefore, DNA amplification can be completed in a single step by incubating the mixture of primers with polymerase and template in a reaction buffer using a simple heat block or water bath^18^. It can typically produce results with high yield and specificity in less than 30 min at a single temperature. These features make the technique rapid and portable which further add to the purpose of on-field applications using simple devices. LAMP technique is known to be well-suited for several molecular diagnostic applications ranging from the laboratory and POC screening of infectious agents for various diseases, food testing, environmental testing, etc.^19–21^. Diagnostic kits and techniques using LAMP have been developed for rapid detection of several difficult pathogenic agents for highly infectious diseases like tuberculosis^22^, leishmaniasis^23^, dengue^24^, HIV/AIDS^25^, zika virus disease^ 26^, salmonellosis^27^, etc*.* More importantly, the advent of LAMP has led to improved diagnosis of neglected tropical diseases all over the world^28–31^.

The laboratory detection method for any nucleic acid amplification technique is generally post-reaction agarose gel electrophoresis. However, for on-field, simple, rapid and user-friendly endpoint detection, various amplicon detection methods have been explored by researchers that can be combined with LAMP technique to produce immediate and easy to read results. Esmatabadi et al.^32^ has reviewed a variety of detection techniques such as colorimetry, turbidometry, hybridization probes, lateral flow dipsticks, ELISA, functionalized gold nanoparticles, etc. that have potential to be combined with LAMP method for easy detection of amplification products. The objective of the present study was to make the ML detection technique rapid, easy to read as well as affordable. Therefore, different detection techniques like colorimetry, turbidity evaluation and bridge flocculation methods have been used for detection of LAMP amplification products.

Since, ML is uncultivable in vitro and possesses a highly reduced genome that has a high degree of identity with MTB genome, specific LAMP technique for ML detection has not been forthcoming, despite its innately unique benefits as a diagnostic tool for disease detection in poor and remote areas of the world. To our knowledge, current study is the first report on presenting LAMP as an efficient diagnostic tool for ML.

## Methods

### Institute approvals

This work was carried out with proper approval from the Institutional Ethics Committee (IEC) of Jamia Hamdard, New Delhi (Reference No. 3/19 dated March 14, 2019). Blood samples were obtained from leprosy patients after obtaining written informed consent at HAHC Hospital, Jamia Hamdard, New Delhi. Sample collection protocols and all methods in the study were performed with strict adherence to the guidelines and regulations of IEC, Jamia Hamdard, New Delhi. Work was also approved by Institutional Bio-Safety Committee (IBSC), Jamia Hamdard, New Delhi (Reference No. 3.1 dated May 15, 2019).

### Materials

QIAamp Blood mini kit (Qiagen, India) was used for DNA extraction from clinical blood samples. *Bacillus stearothermophillus* DNA Polymerase 3.0 (New England Biolabs, UK) and primers (Integrated DNA Technologies, USA) were used for the nucleic acid amplification. *Escherichia coli* (strain MG1655, JH-Institute of Molecular Medicine, Jamia Hamdard, New Delhi) was used as double negative control. MTB DNA (strain H37RV, National Institute of Immunology, New Delhi) was used as negative control. Ethidium bromide (Sigma-Aldrich, USA), propidium iodide (Merck, USA) and SPRI (solid phase reversible immobilization) magnetic beads (Canvax, Spain) were used for detection of amplification products. All other chemicals, unless otherwise specified, were from Sigma-Aldrich (USA).

### DNA Extraction

ML DNA was extracted from SSS samples of leprosy-positive subjects using the Proteinase K method^33^. Concentration of the extracted DNA was measured using microvolume spectrophotometer (NanoDrop 3300). Blood samples from patients were obtained in EDTA tubes and 300 μl of blood samples was used for genomic DNA extraction. DNA of *E.coli* was extracted using phenol–chloroform method as described by F. He with slight modifications^34^.

### LAMP target selection and primer designing

LAMP assay utilizes three sets of primers viz. forward and backward outer primers (F3, B3), inner primers (FIP, BIP) and loop primers (LF, LB). These promote formation of ‘hairpin-like’ loop structures at the start of the reaction, making way for high degree of self-priming for DNA synthesis and the reaction becomes self-sustaining, producing robust template amplification^35^. For determining ML target sequences for primer designing, available literature was searched for DNA amplification methods, mainly PCR. Several gene sequences such as RLEP and its subtypes (RLEP2, RLEP3, RLEP4), 18-kDa, 16S rRNA, SodA, rpoT, Pra, etc. have been reported and used by researchers to design primers for PCR detection of ML^36–44^. As per the literature available, isothermal technique has not yet been used for DNA amplification for detection of ML DNA. RLEP, a short repetitive sequence of only 129 bp, has shown high specificity for ML detection amongst all PCR-based studies. But due to its extremely short length it is a difficult target for designing six primers required for LAMP technique and appropriate primers could not be obtained by any primer designer software. We therefore searched other gene targets using LAMP primer designer software (Optigene version 1.12, UK) and the selected primers are enlisted in Table 1.

**Table 1:** List of LAMP primers used for detecting ML.

Gene	Primer	Sequence
RLEP2	R3F3	CTTTACCCGATAGCAGCG
	R3B3	ATTCTTAAGCGAAGAGGAGC
	R3FIP	GTCGGATTCTAGGCGGTGCCTGTCAACGCGATACTTCA
	R3BIP	GTCAGCTCGGGTAAGAACGATCACCATTCAGTGCGTGATC
	R3LF	CTCAGTGAGCCGTCTTGG
	R3LB	CCAAACACCACACGATGTC
RLEP3	R3F3	CTTTACCCGATAGCAGCG
	R3B3	ATTCTTAAGCGAAGAGGAGC
	R3FIP	GTCGGATTCTAGGCGGTGCCTGTCAACGCGATACTTCA
	R3BIP	GTCAGCTCGGGTAAGAACGATCACCATTCAGTGCGTGATC
	R3LF	CTCAGTGAGCCGTCTTGG
	R3LB	CCAAACACCACACGATGTC
RLEP4	R4F3	AACACCACCCAAACCAAA
	R4B3	CTACCAGGAGGTAGGTAACC
	R4FIP	TGCGGTATTGGTGCCAGACCGACGACACCTTGAAGTC
	R4BIP	GGTAGACGAGGCTCTAGGTGTCATACGGCTGTACTTGGAC
	R4LF	CAGTAACCAGTAACTTGTCCGT
	R4LB	ACTGGTAGCTCGTCCTGG
18-kDa	18KF1	ACATGCTGATGCGTACTGAC
	18KR1	TGTTTATGGTCTGGTGTCCG
	18KF2	TGATGCGTACTGACCCGTTCC
	18KR2	TTTTGTTTATGGTCTGGTGTCCG
SodA	SAF3	CGCTGGAACCACATATCTC
	SAB3	TTCATCAATGTCAGTGGCTAG
	SAFIP	GGCAAGCGCGTCATTGACGATCAACGAGATCCACCAC
	SABIP	GACGACCACTCCGCGATTTCAGATGGAGTGGTTGACG
	SALF	CTTTGACATATGCGGCGTG
	SALB	TTCTGAACGAGAAGAACCTGG
16S rRNA-N	16F3	ACGTCAAGTCATCATGCC
	16B3	GGAACGTATTCACCGCAG
	16FIP	TCGCTTAACCTTGCGGCATTATGTCCAGGGCTTCACA
	16BIP	CGGTCTCAGTTCGGATCGGCGTTGCTGATCTGCGATTA
	16LF	GTACCGGCCATTGTAGCA
	16LB	GTGAAGTCGGAGTCGCTAG
16S rRNA-S	16SF3	CGAACGGAAAGGTCTCTAAA
	16SB3	GTCGCTGCATCAGGCTTG
	16SFIP	CCACCAACAAGCTGATAGGCCGGATAGGACTTCAAGGC
	16SBIP	TACCAAGGCGACGACGGGTACGCCCATTGTGCAATATTC
	16SLF	TTCCACCACAAGACATGC
	16SLB	CACACTGGGACTGAGATACG

### Nucleic acid amplification and optimization

Detailed LAMP amplification technique is described by Nagamine et al.^18^. Briefly, 25 μl reaction mixture contained 0.2 μM outer primers (F3 and B3), 0.4 μM loop primers (LF and LB), 0.8 μM inner primers (FIP and BIP), optimized reaction buffer (20 mM Tris–HCl, 10 mM (NH_4_)_2_SO_4_, 150 mM KCl, 2 mM MgSO_4_, 0.1% Tween 20; pH 8.8), 200 nM dNTPs, 8 U *Bacillus stearothermophilus* DNA polymerase I and 2 μl template DNA. The reaction was incubated at 66 °C for 1 h and amplicons were initially analyzed by agarose gel electrophoresis using ethidium bromide (EtBr) staining. The method was optimized for reaction conditions such as reaction duration, temperature and heat source for the most efficient amplification conditions.

### LAMP sensitivity and specificity

LAMP was performed using serial dilutions of template DNA to evaluate the sensitivity. The specificity of LAMP primers was determined by using MTB and *E. coli* DNA as negative controls to nullify any chance of cross reactivity.

### Comparison with conventional method

To determine the rapidity and efficiency of LAMP as a nucleic acid amplification technique for ML detection, a comparison was made with conventional PCR method using protocol and PCR primers described by Donoghue et al*.* and Truman et al*.*^38, 45^. In brief, primers for ML-specific RLEP gene (RF: 5′ TGCATGTCATGGCCTTGAGG 3′, RR: 5′ CACCGATACCAGCGGCAGAA 3′) along with PCR reagents (reaction buffer, Taq polymerase, dNTPs) and samples were mixed and incubated in a thermocycler (Bio Rad, USA) with cycling conditions as follows: initial denaturation at 95° C for 10 min followed by one cycle of 94° C for 2 min, 58° C for 2 min and 72° C for 2 min. This was followed by 40 cycles of 94° C for 30 s, 60° C for 30 s and 72° C for 45 s. Final extension was done at 72° C for 10 min. The amplicons were visualized by 2% agarose gel electrophoresis.

### Validation using clinical samples

Blind studies were done using random blood samples from eighteen ML and MTB infected patients to validate the LAMP method of detection. Of these, four were females and fourteen were males. Samples from patients were taken before the start of any therapy. Diagnosis was made based on their symptoms, microscopic detection and SSS test. Children and pregnant ladies were excluded from the study. DNA from blood samples were isolated using DNA extraction kit and LAMP was performed along with controls. Results were analyzed using EtBr-based gel electrophoresis.

### Statistical analysis

Based on observations and outcomes, the sensitivity, specificity, positive predicted value (PPV) and negative predicted value (NPV) of clinical studies were calculated to determine the efficiency of LAMP method for ML detection.

### Visual detection methods

Rapidity of a technique includes fast and convenient assessment of results. User-friendly visual detection methods for qualitative determination of DNA amplification methods were used apart from time-intensive conventional gel electrophoresis.

#### Colorimetric detection

DNA intercalating dye propidium iodide was used to differentiate between positive and negative reaction samples 1 μl of 1 mg/ml stock solution of the dye was used for each reaction mixture after amplification and incubated at room temperature for 1 min before visualizing under UV transilluminator.

#### Visual detection of turbidity

Visual as well as spectroscopic detection of turbidity which is caused by release of free pyrophosphates during DNA multiplication was performed using increasing concentrations of magnesium ions in solution, as described by Esmatabadi et al*.*^32^.

#### Bridge flocculation assay

This method employs use of SPRI (solid phase reversible immobilization) beads where amplicons, if present, bind with the nanosized-magnetic beads and form a pellet, in contrast to the dispersed beads in the absence of amplified products^46^. The procedure was followed as described by Benjamin et al.^47^ with slight modification. 5 μl of amplified product was mixed and incubated with 1.5–1.8 times (v/v) bead solution at room temperature for 5 min. After magnetic separation of beads and 80% ethanol wash, 30 μl of flocculation buffer (100 mM sodium acetate and 1% v/v Tween20, pH 4.4) was added to the beads. Tubes were observed for flocculation immediately.

## Results

### LAMP primer design

**Figure 1:**
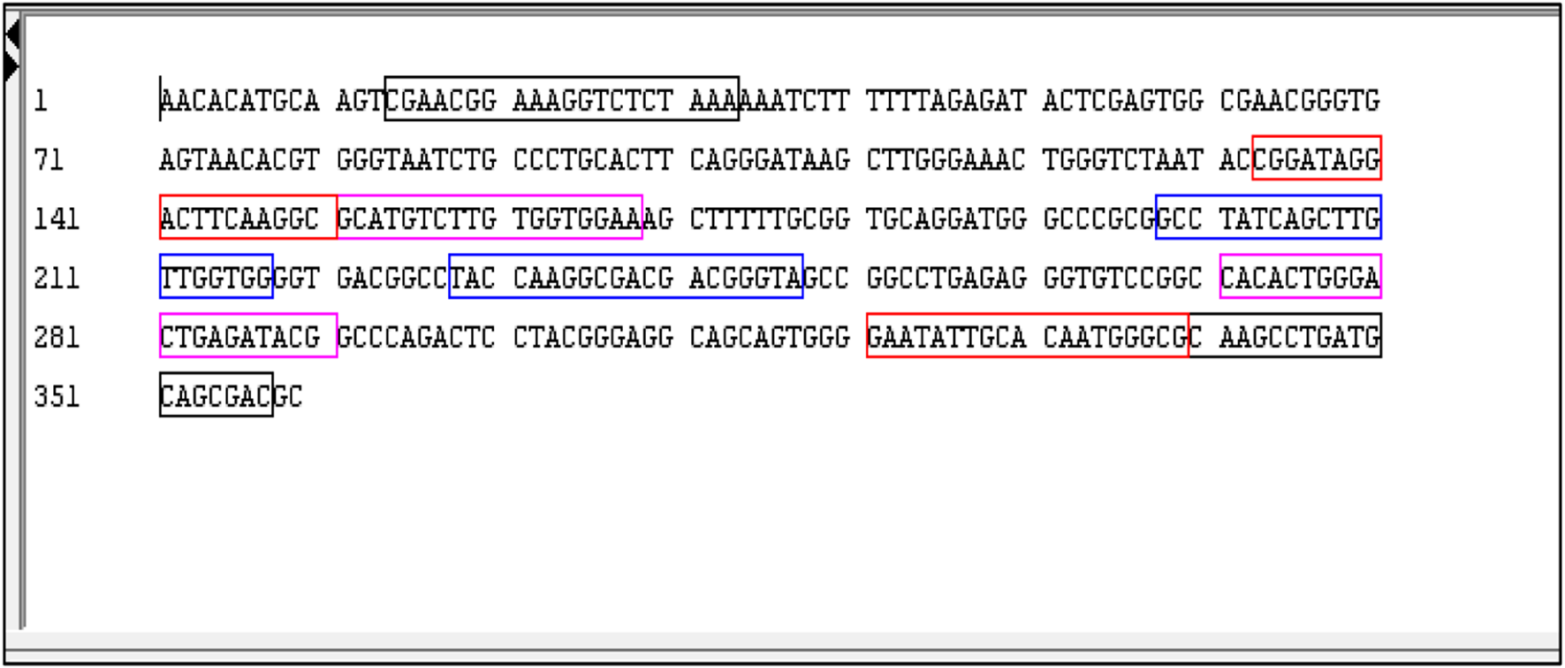
Location of 16S rRNA-S LAMP primers in target gene sequence.

To identify ML- specific LAMP primers, we designed primers for 16S rRNA and other genes (Table 1). None of the listed primers except those for 16S rRNA gave discernible template amplification. Therefore, we used the 16S rRNA primers for detection of ML. However, 16S rRNA-N primers showed amplification with both ML and MTB DNA samples, showing that these primers are not specific for ML. To overcome this, Basic Local Alignment Search Tool (NCBI BLAST) analysis of 16S rRNA gene of ML (GenBank Accession No. X53999) was done and the sequence was aligned with MTB 16S rRNA gene sequence (GenBank Accession No. NR_102810). Mismatched regions between the 16S rRNA sequences of both species were identified and these sequences were used to design specific LAMP primers (3 sets) for ML (16S rRNA-S: Table 1), using Optigene LAMP designer 1.15 software. Bioinformatic analysis of the targeted sequence showed no overlap with any other Mycobacterial species sequence. These primers selectively amplified ML, but not MTB, DNA thereby providing the distinguishing specificity to be used for further studies. Figure 1 shows the location of the 16S rRNA-S LAMP primers in the 16S rRNA genomic sequence.

### Optimization of LAMP parameters

Optimization of the LAMP method was done by varying the reaction conditions to identify a set of conditions which would provide robust amplification signals, as described below.

**Figure 2:**
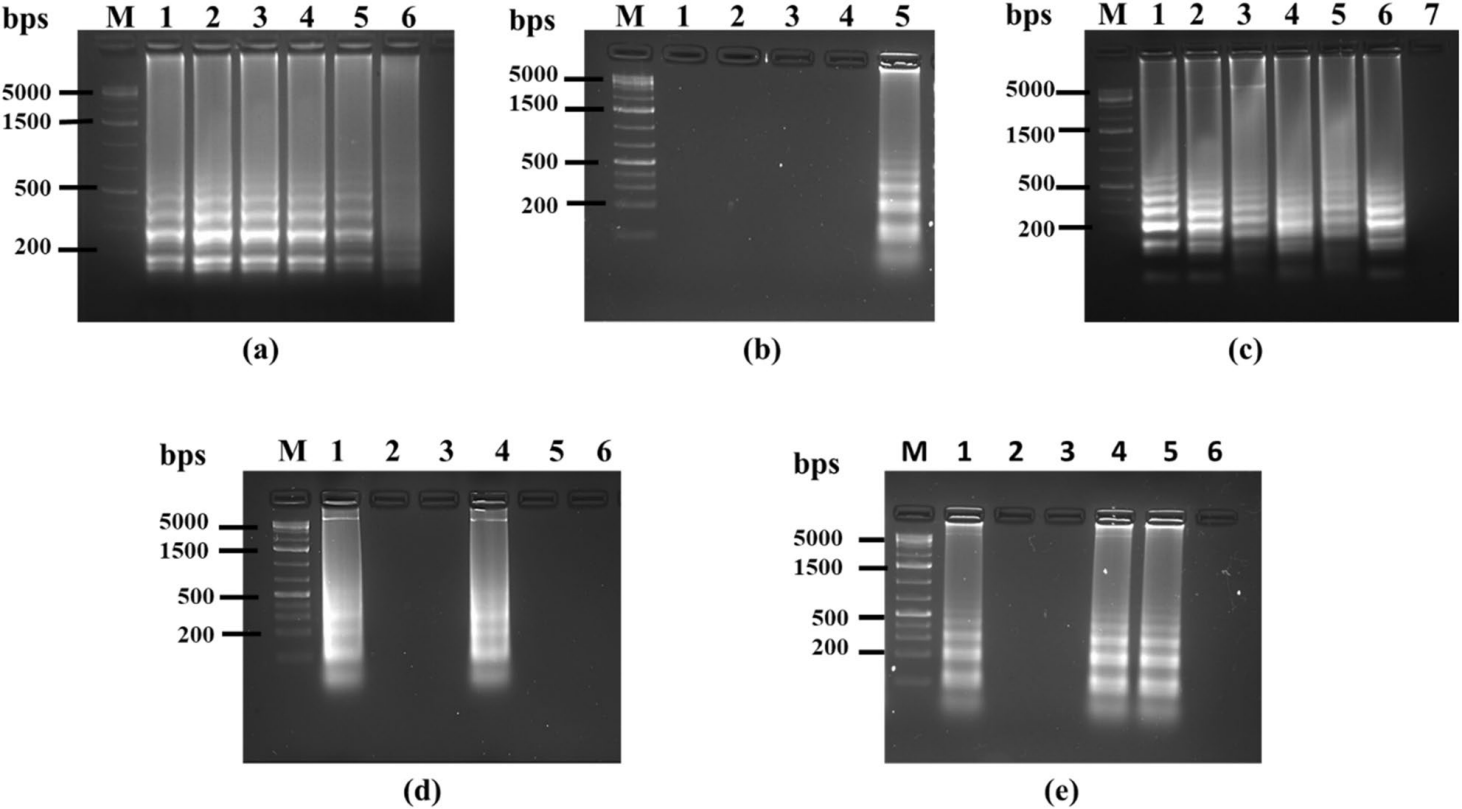
Gel electrophoresis analysis of amplification reactions under different LAMP parameters. (**a)** Temperature optimization. Lanes: (M) Ladder, (1) 65 °C, (2) 66 °C, (3) 67 °C, (4) 68 °C, (5) 69 °C and (6) 70 °C (**b**) Reaction time optimization. Lanes: (1) 0 min, (2) 15 min, (3) 30 min, (4) 45 min and (5) 60 min, (**c**) Template concentration optimization. Lanes: (1) 1 ng, (2) 0.5 ng, (3) 0.1 ng, (4) 0.05 ng, (5) 0.01 ng, (6) 5 pg, (7) 1 pg, (**d**) Comparison of reaction heat source. Lanes 1–3: Heat block, Lanes 4–6: Water bath. (Lanes 1 & 4) ML DNA, (Lanes 2 & 5) MTB control, (Lanes 3 & 6) *E.coli* control, Lane M ladder, (**e**) Specificity of 16S rRNA-S primers. Lanes 1–3: 16S rRNA-S primers, Lanes 4–6: 16S rRNA-N primers. (Lanes 1 & 4) ML DNA, (Lanes 2 & 5) MTB control, (Lanes 3 & 6) *E.coli* control, Lane M is ladder.

- *Reaction temperature*: LAMP reactions were performed at different temperatures (65° to 70 °C) to determine the optimum temperature necessary for the amplification. As shown in Fig. 2(a), beyond 66 °C amplification efficiency was greatly reduced, followed by no amplification at all at 70 °C. The temperature associated with highest amplification signal i.e. 66 °C was chosen for our method.
- *Reaction time*: LAMP was carried out for different incubation periods (0 min, 15 min, 30 min, 45 min and 60 min) to specify the shortest time for amplification. After every time point, the reaction was ceased by denaturing the polymerase at 80 °C for 2 min. Figure 2(b) shows that 1 h reaction time is the minimum period for LAMP amplification.
- *Determination of sensitivity*: The sensitivity of the method was evaluated by using various concentrations of template DNA for LAMP reactions. Lowest detectable DNA concentration governs the sensitivity of the method. The results, as indicated in Fig. 2(c), show the detection limit in picograms, with 5 pg/μl being the lowest concentration showing strong amplification. This anchors the robustness of the technique towards early diagnosis of ML infection even with low bacterial DNA load.
- *Source of heating*: Assays were performed using different thermal sources viz. heat block and water bath, to identify the feasibility of the assay in poor resource settings. Figure 2(d) shows the comparison of heat sources depicting similar amplification results in both cases.
- *Specificity determination*: The main aim of our study was to develop a LAMP detection method specific to ML to avoid cross-detection of MTB, a common and persistent technical obstacle. Our LAMP primers did not show any amplification signal with MTB DNA (Fig. 2e). Even at concentrations of 20 ng/μl of template MTB DNA, no amplification was observed, confirming the high degree of specificity of our ML-specific primers. Cross reactivity for primers was tested using *E. coli* as double negative control in each study.

### Standardization of intensity vs. concentration

**Figure 3:**
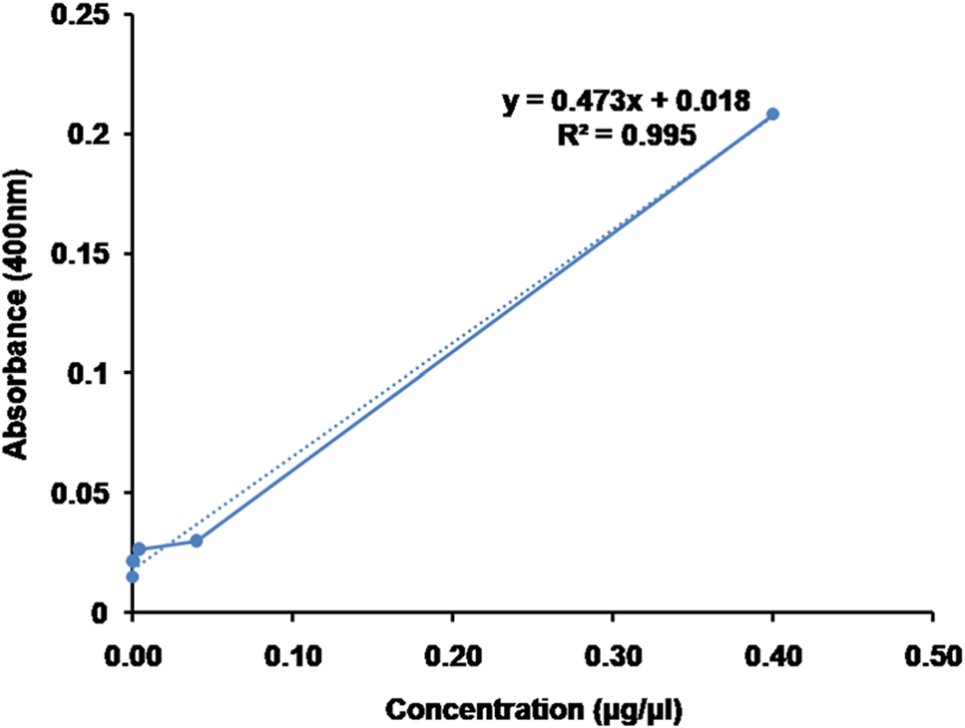
Calibration curve of amplification intensity in terms of absorbance at different input template concentrations.

The intensity of amplification was detected by spectrophotometrically measuring absorbance of tenfold serial dilutions of amplified DNA at 400 nm. A calibration curve (Fig. 3) was obtained showing increasing amplification intensity with rise in DNA concentration. The R^2^ value of the curve was determined to be 0.995. Linear curve fitting was done to fit the amplification intensity response on increasing the input concentration. The equation for the best fit line was x = [(y − 0.018)/0.473] where x represents the input DNA concentration and y represents the absorbance at 400 nm, indicating the intensity of amplification.

### Evaluation of LAMP technique in clinical samples

**Figure 4:**
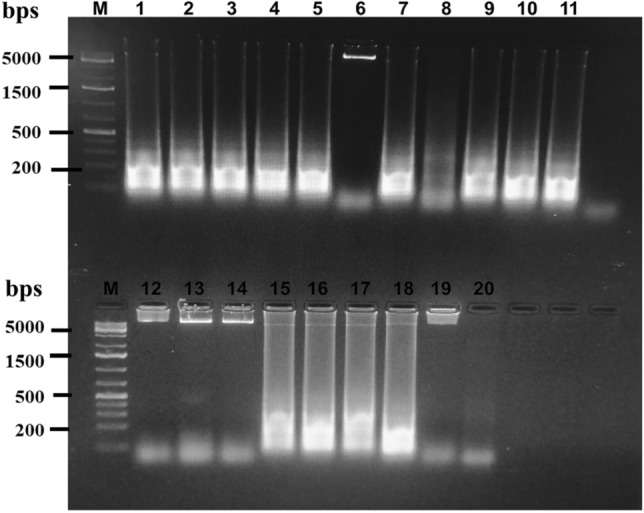
ML-specific LAMP technique in clinical samples. Lane 1: Positive control, Lanes 2–11 and 13–20: Clinical samples, Lanes 12 and 21: No template controls.

To validate the technique in clinical samples, blind studies were performed using random blood samples from patients diagnosed with either ML or MTB. Blood samples were selected for evaluation of this assay instead of skin smears so as to make this technique less painful and more convenient. Eighteen clinical samples were assessed with the LAMP technique and the results were cross-validated with the conventional detection method, as described in Materials and Methods. DNA extracted from blood samples, along with positive and negative controls, were screened for LAMP amplification (Fig. 4). Around twelve samples were found to be positive and six negative for ML by LAMP method. The same samples were tested by conventional method and the observation details along with results are given in Table 2. The results show complete diagnostic concurrence between LAMP technique and conventional technique, with 100% clinical specificity. The PPV and NPV too was 100% in LAMP as compared with conventional method.

**Table 2:** Comparative analysis of conventional method and LAMP detection of ML.

LAMP	Conventional Method Positive	Conventional Method Negative	Total	Sensitivity (%)	Specificity (%)	PPV (%)	NPV (%)
Positive	12	0	12	100	100	100	100
Negative	0	6	6	–	–	–	–
Total	12	6	18	–	–	–	–

### Comparison of LAMP with regular PCR

**Figure 5:**
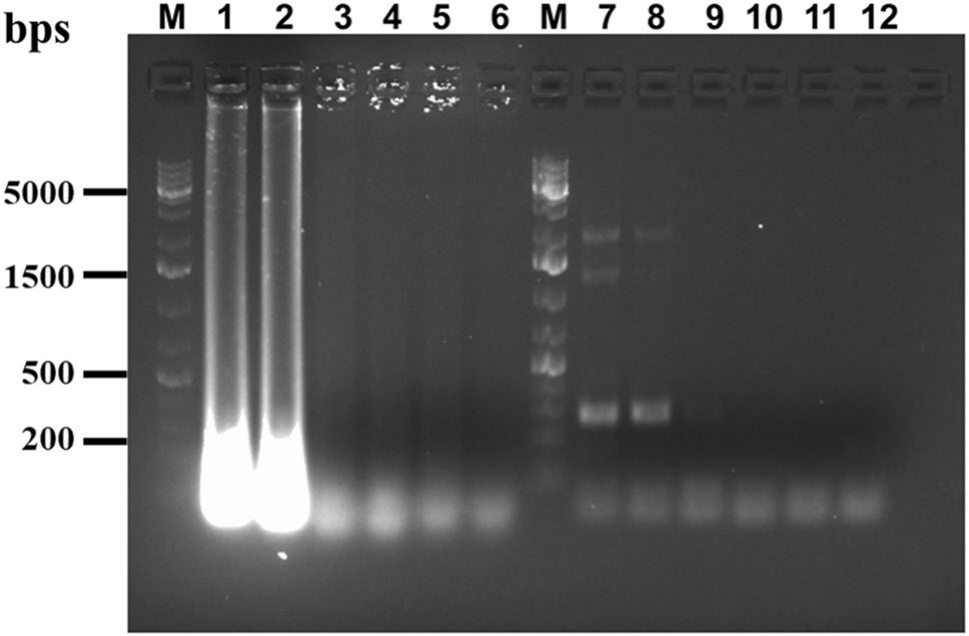
Comparative analysis between LAMP and regular PCR. Amplification was done using positive control and a suspected clinical sample by LAMP in lanes 1 and 2 and by PCR in Lanes 7 and 8, respectively. Others are negative controls viz*.* MTB control (Lanes 3 and 9), *E.coli* control (Lanes 4 and 10), no primer (Lanes 5 and 11) & no template (Lanes 6 and 12), Lanes L and M are ladders.

So far, PCR has been seen as the only nucleic acid amplification technique available for molecular diagnosis of ML. We compared the efficiency and sensitivity of our LAMP technique with traditional PCR method. Figure 5 shows that LAMP gave similar results as regular PCR and, in fact, better visual bands in 60 min using a simple water bath. In contrast, PCR took more than two and a half hours using a thermocycler for detectable bands. This implies that LAMP is more efficient with regards to rapidity, resource utilization, and ease of detection as compared to conventional PCR method.

In recent years, several PCR methods with varying sensitivities and specificities using different target sequences have been published. A side-by-side comparison was made (Table 3) between some of those published studies with our LAMP technique of detection. It can be noted that LAMP has the quickest turnaround time to amplify the bacterial DNA in clinical blood samples and has eliminated the risk of false positives or false negatives. In Table 3, many of the references have used biopsy samples which are painful to collect and take 2 h or more for target amplification. Moreover, they were unable to achieve high sensitivity and specificity in their studies. The method by Beissner et al. reported comparable sensitivity and specificity but it used a combination of two techniques which made the procedure more complex and cost- and time-intensive. Therefore, it can be concluded that the LAMP method has the potential to be developed for clinical use for its rapidity, portability, high sensitivity and specificity.

**Table 3:** Comparison of LAMP with PCR-based techniques.

Method	Sample	Target gene	Amplification time	Sensitivity/ Specificity	Test on clinical samples	Proposed By
LAMP	Blood	16S rRNA	60 min	100% / 100%	Yes	-
Conventional PCR	Nerve biopsies	RLEP	2 h	92.3% / 54.5%	Yes	Tiwari et al.^48^
Multiplex PCR	Skin biopsies	ML1545, ML2180, ML2179	2hours 45 min	75.61% / 100%	Yes	Chaitanya et al.^49^
qPCR	Skin biopsies	RLEP	1 h 45 min	84% / 100%	Yes	Azevedo et al.^50^
RT-PCR	Nasal swab	RLEP/ 16S rRNA	1 h 45 min	100% / 100%	Yes	Beissner et al.^9^

### Methods for detection of LAMP amplicons

**Figure 6:**
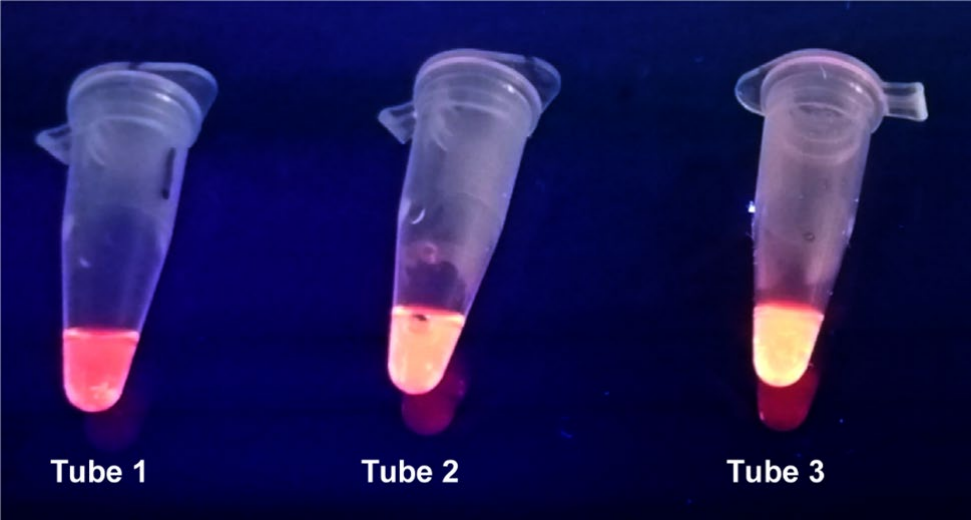
Propidium iodide detection of LAMP amplicons. Bright pink fluorescence (Tube 1) shows presence of amplified ML DNA in sample while negative samples, MTB control (Tube 2) and No template control (Tube 3), give orange fluorescence.

**Figure 7:**
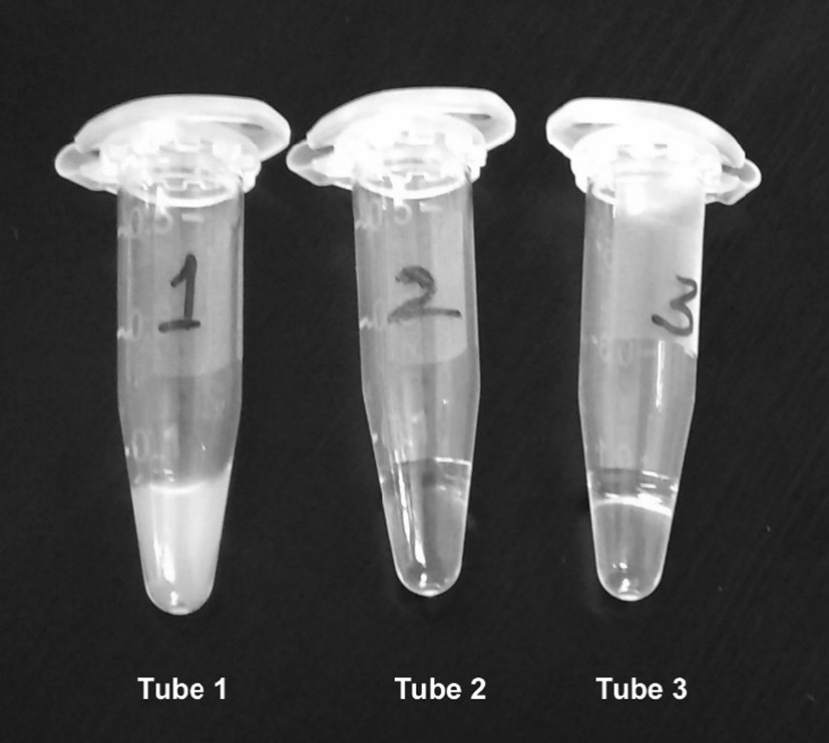
Visual turbidity detection of LAMP amplification. Clear negative samples (Tube 2: MTB control and Tube 3: No template control) can be visually differentiated from the positive sample (Tube 1: ML DNA) showing turbidity.

**Figure 8:**
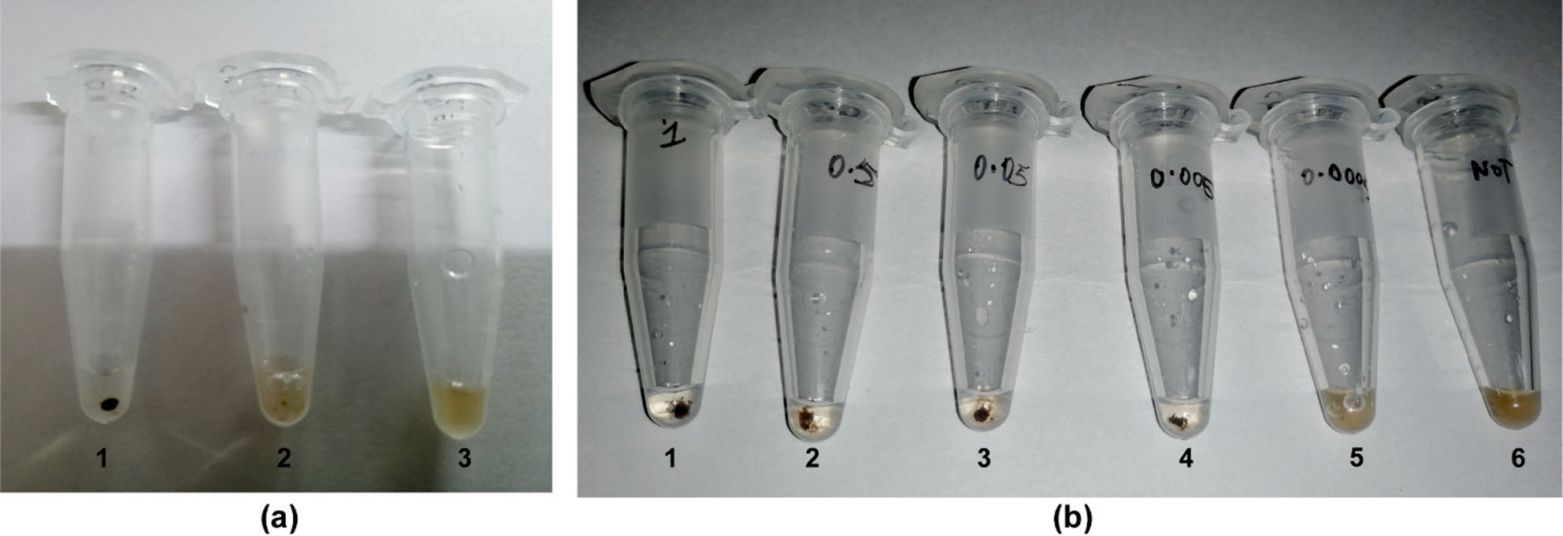
Detection by bridge flocculation method. (**a**) Visual flocculation of amplified product. Positive sample (1) shows distinct flocculation whereas MTB control (2) and No template control (3) appear brown with no flocculation (**b**) Sensitivity determination of flocculation method. Different dilutions, 1 ng/μl (1), 0.5 ng/μl (2), 0.05 ng/μl (3), 5 pg/μl (4), 0.5 pg/μl (5) template DNA were used for LAMP and bridge flocculation detection alongwith No template control (6).

- *Colorimetric detection:* Apart from conventional gel electrophoresis for detecting DNA amplification, other detection methods for visual identification of amplified products were also used in order to enhance the ease and cost of operation. Propidium iodide was used to qualitatively identify positive LAMP amplification reactions without gel electrophoresis. This fluorescent dye intercalates between DNA strands and gives fluorescence under UV wavelength. Figure 6 shows propidium iodide dye detection of LAMP products where positive sample with amplified product fluoresced bright pink while negative samples appeared orange in color.
- *Turbidity visualization*: During the amplification reaction, pyrophosphate ions are produced which, in the presence of magnesium ions, form magnesium pyrophosphate that precipitates in solution. The resulting turbidity can be visually detected. Difference in visual turbidity was observed between positive samples and negative controls (Fig. 7), constituting another basis of visual detection of amplification results.
- *Bridge flocculation method*: This method is generally used to purify DNA amplicons by using SPRI magnetic beads. But here, it is re-purposed as a detection technique to determine the presence of LAMP amplification products. In the presence of magnetic field, the beads aggregate and amplified DNA get entangled with the beads forming floccules which prevent their re-dispersion after removal of the magnetic field. In the absence of amplification, beads re-disperse in the solution after removal of the magnetic field. Figure 8(a) shows the visual difference between positive and negative LAMP reactions using SPRI beads. Sensitivity of this detection method was also determined by using different dilutions of DNA for LAMP amplification and performing bridge flocculation assay for detection of products. Figure 8(b) shows visible flocculation with as low as 5 pg/μl template concentration. This shows the potential of this detection assay in combination with LAMP technique for easy and rapid point-of-care detection of ML DNA.

## Discussion

Early and accurate detection of ML is crucial not only for proper treatment regimen but also to lower the burden and transmission in endemic areas, which in almost all cases are in socioeconomically-deprived and access-limited regions of the world. Furthermore, specificity of ML detection is often compromised due to the presence of homologous sequences in other Mycobacterium species, most notably MTB. We report the development and standardization of a rapid, sensitive and economical method of detecting ML DNA using an optimized LAMP technique. Primers were designed that specifically amplified target regions of 16S rRNA gene of ML these primers could differentiate ML from closely-related MTB samples, forming the basis of a sensitive and specific diagnostic tool. Blind studies using 18 clinical samples, including both positive and negative samples, established the efficiency and robustness of the technique with 100% sensitivity, specificity, PPV and NPV. An intrinsic methodical limitation of this study is the small number of clinical samples used to validate our in vitro results. Although the challenges in identification and recruitment of patients in early stages of ML infection remain high, expanding the clinical sample size in future studies will help in eliminating any possible bias or inaccuracy in our proposed technique. The technique is suitable for detecting ML DNA even at picogram concentrations, making it highly sensitive to low bacterial load and can be applied for early diagnosis of the disease before any permanent physical mutilation. It has an additional advantage of having a short turnaround time to produce results using regular bench equipments like water bath or heat block. Point-wise analysis of our LAMP technique with prevalent and published conventional PCR methods indicates that this technique shows equivalent, if not enhanced, sensitivity for ML genomic DNA detection. Additionally, this technique can be combined with rapid detection methods like colorimetry, turbidometry and bridge flocculation assay for naked-eye visualization of positive reaction products, further making this diagnostic technique for point-of-care use easily readable and user-friendly. In resource-poor settings where rapid, inexpensive and on-site testing is required to detect the potential source of infection and transmission, this method could be utilized for improved and simplified monitoring of potential leprosy patients at early stages of infection. Besides early diagnosis of infection, this technique may prove to be useful in monitoring the reduction in bacterial load during the course of multi-drug therapy in infected patients. Also, this technique could be applied for transmission studies to investigate human-to-human transmission in household and community contacts.

Currently, leprosy is preliminarily diagnosed through observation of physical manifestations and slit skin smear test. Apart from being painful, skin biopsy-based tests are lengthy, lack sensitivity, require an equipped lab and trained personnel and often result in false positives or false negatives. Sophisticated molecular diagnostic techniques like quantitative real-time PCR are not suitable alternatives for simple, cheap and reliable on-field testing tools. Unlike most other pathogens, detection of ML is difficult due to the fact that the bacillus cannot be cultured in vitro. The LAMP technique for ML detection using blood samples and readily available, simple instruments alongside quick visible detection methods we have described in this study, has the scope to be further developed as a point-of-care testing unit in field settings that can complement or even replace traditional diagnostic methods.
